# Inhibition of Enterovirus 71 (EV-71) Infections by a Novel Antiviral Peptide Derived from EV-71 Capsid Protein VP1

**DOI:** 10.1371/journal.pone.0034589

**Published:** 2012-05-01

**Authors:** Chee Wah Tan, Yoke Fun Chan, Kooi Mow Sim, Eng Lee Tan, Chit Laa Poh

**Affiliations:** 1 Department of Medical Microbiology, Faculty of Medicine, University of Malaya, Kuala Lumpur, Malaysia; 2 Department of Chemical Science, Faculty of Science, Universiti Tunku Abdul Rahman, Kampar, Malaysia; 3 Centre for Biomedical and Life Sciences, Singapore Polytechnic, Singapore, Singapore; 4 School of Health and Natural Sciences, Sunway University, Petaling Jaya, Malaysia; University of Kansas Medical Center, United States of America

## Abstract

Enterovirus 71 (EV-71) is the main causative agent of hand, foot and mouth disease (HFMD). In recent years, EV-71 infections were reported to cause high fatalities and severe neurological complications in Asia. Currently, no effective antiviral or vaccine is available to treat or prevent EV-71 infection. In this study, we have discovered a synthetic peptide which could be developed as a potential antiviral for inhibition of EV-71. Ninety five synthetic peptides (15-mers) overlapping the entire EV-71 capsid protein, VP1, were chemically synthesized and tested for antiviral properties against EV-71 in human Rhabdomyosarcoma (RD) cells. One peptide, SP40, was found to significantly reduce cytopathic effects of all representative EV-71 strains from genotypes A, B and C tested, with IC_50_ values ranging from 6–9.3 µM in RD cells. The *in vitro* inhibitory effect of SP40 exhibited a dose dependent concentration corresponding to a decrease in infectious viral particles, total viral RNA and the levels of VP1 protein. The antiviral activity of SP40 peptide was not restricted to a specific cell line as inhibition of EV-71 was observed in RD, HeLa, HT-29 and Vero cells. Besides inhibition of EV-71, it also had antiviral activities against CV-A16 and poliovirus type 1 in cell culture. Mechanism of action studies suggested that the SP40 peptide was not virucidal but was able to block viral attachment to the RD cells. Substitutions of arginine and lysine residues with alanine in the SP40 peptide at positions R3A, R4A, K5A and R13A were found to significantly decrease antiviral activities, implying the importance of positively charged amino acids for the antiviral activities. The data demonstrated the potential and feasibility of SP40 as a broad spectrum antiviral agent against EV-71.

## Introduction

Enterovirus 71 (EV-71) belongs to the Human Enterovirus A species of the genus Enterovirus within the family *Picornaviridae* [1]. EV-71 is composed of a single-stranded, positive-sense RNA of approximately 7411 nucleotides enclosed within an icosahedral capsid assembled from 60 copies of each of the four structural proteins, VP1–VP4 [2].

EV-71 is one of the main etiological agents of hand, foot and mouth disease (HFMD) which is generally regarded as a mild childhood disease. HFMD is characterized by the development of mild febrile illness with papulovesicular lesions on the hand, foot and mouth. Several epidemics with high mortalities have occurred in Europe in the 1970s (Bulgaria 1975 and Hungary in 1978) [3],[4]. However, in recent years, it has emerged as a pathogen capable of causing severe neurological complications such as brain stem encephalitis and acute flaccid paralysis in infants and young children (<6 years old) in Asia [5]–[7]. The impact of high fatalities and long-term neurological sequelae in severely infected children isolated from large scale HFMD outbreaks in Malaysia (1997),Taiwan (1998) and China (2009) indicated that EV-71 should be regarded as the most feared neurotrophic enterovirus after the eradication of poliovirus [8]–[10]. Currently, there is no vaccine for prevention or antiviral to treat EV-71 infections [6], [11]. Thus, there is a need to develop better and effective antiviral agents to treat future EV-71 infections.

Peptides that can block viral attachment or entry into host cells have therapeutic potentials. Enfuvirtide is the first peptide-based inhibitor of viral fusion approved by the FDA in March, 2003 for clinical use. Enfuvirtide is a 36-amino acid peptide derived from the HR2 sequence of the transmembrane protein gp41 of HIV-1 and is the prototype of new antivirals [12], [13]. Recent studies have discovered potential antiviral peptides against Hepatitis C by screening 441 overlapping peptides (18-mers) covering the entire HCV polyprotein [14] and a peptide derived from the pre-S1 surface protein of Hepatitis B virus was able to exhibit antiviral properties against Hepatitis B virus infection [15]. Shih *et al.* (2004) showed that BPROZ-194 binds to VP1 and was effective in inhibiting viral attachment or viral uncoating. This indicated that VP1 is a good target to derive potentially antiviral peptide sequences [16].

In the present study, 95-overlapping peptides (15-mers) covering the entire EV-71 capsid protein, VP1, were chemically synthesized. These peptides were screened for their ability to inhibit EV-71 infection in Rhabdomyosarcoma (RD) cells. Four peptides were found to inhibit EV-71 infection by more than 80% when screened at 100 µM. One peptide, SP40, was selected for further studies as it was able to inhibit EV-71 infection at 89% in RD cells and it mimics the sequence that is highly conserved in all EV-71 genotypes.

## Materials and Methods

### Cell and Viruses

Rhabdomyosarcoma (RD, ATCC # CCL-136) cells, African green monkey kidney (Vero, ATCC # CCL-81) cells, human cervical adenocarcinoma Epithelial (HeLa, ATCC # CCL-2) cells and human colon adenocarcinoma (HT-29, ATCC # HTB-38) cells were obtained from American Type Culture Collection (ATCC, USA). RD cells were grown in Dulbecco’s Modified Eagle’s Medium (DMEM) supplemented with 10% fetal bovine serum (FBS). Vero and HeLa cell lines were grown in Eagle Minimal Essential Medium (EMEM) supplemented with 10% FBS. HT-29 cells were grown in McCoy’s Medium supplemented with 10% FBS. EV-71 strain 41 (5865/SIN/000009) (GenBank accession number: AF316321), SHA66/97 (GenBank accession number: AM396586); BrCr (GenBank accession number: AB204852) and SHA52 (GenBank accession number: AM396584) were propagated in RD cells supplemented with 2% FBS. Coxsackievirus A16 (strain 22159) and poliovirus type 1 used were clinical isolates and were propagated in RD and Vero cells supplemented with 2% FBS, respectively.

### Peptides

A set of 95 overlapping synthetic peptides spanning the entire sequence of the VP1 capsid protein of Enterovirus 71 strain 41 (GenBank accession no. AF316321) was synthesized by Mimotopes Pty Ltd. (Clayton Victoria, Australia). Each peptide contains 15-amino acid residues with 12 residues overlapping with the adjacent peptides. All the peptides were reconstituted in 100% dimethyl sulfoxide (DMSO) and stored at −80°C. The peptide stock solution was diluted to the final concentration of 100 µM in the maintenance medium for initial screening. The final concentration of the DMSO was less than 1%. The peptide that showed inhibition of cytopathic effects was identified and synthesized in larger amounts with >95% HPLC purity grade.

### *In vitro* EV-71 Inhibitory Assay of 95-overlapping Synthetic Peptides

Approximately 1.5×10^4^ of RD cells were seeded into each well of a 96-well plate and incubated overnight in a CO_2_ incubator supplemented with 5% CO_2_. Prior to virus infection, EV-71 (100 PFU) were incubated with 100 µM of each synthetic peptide for 1 hour at room temperature with gentle rocking and then transferred to the plate containing RD cells. After adsorption for 1 hour, the inoculum was removed, and the cells were washed twice with the serum free medium. An aliquot of 100 µl of the fresh maintenance medium supplemented with 2% FBS was added and the infected RD cells were incubated at 37°C for 24 hours. After 24-hour post infection, total infectious viral particles were harvested and titrated with plaque assay.

### EV-71 Plaque Assay

The plaque assay was carried out according to Sim *et al.* (2005) with some modifications [17]. In brief, approximately 1.5X10^5^ RD cells were seeded into each well of a 24-well plate, and maintained in the complete growth medium. Prior to viral infection, the complete growth medium was removed. After adsorption for 1 hour, the inocula were removed, and the cells were washed twice, overlaid with 500 µl plaque medium (containing 1.2% carboxymethylcellulose and 2% FBS). After 48 hours of incubation, cells were fixed with 4% formaldehyde and stained with 0.5% crystal violet.

### Inhibition Concentration 50% (IC_50_) Determination

The IC_50_ values of SP40 against EV-71 strains, CV-A16 and poliovirus type 1 were determined using comprehensive assay. In brief, various concentrations of the peptide were prepared and mixed with an equal volume of virus supernatant. The virus-peptide mixtures were then used to infect peptide treated RD cells at a MOI of 0.1. For EV-71, the viral titer was determined by the plaque assay and total viral RNA was quantitated by the RT TaqMan Real-time PCR assay. For CV-A16 and poliovirus type 1, the total infectious viral particles were harvested 24-hour post infection and quantitated by TCID_50_ using Reed and Muench method [18].

### Reverse Transcription (RT) TaqMan Real-time PCR Assay

The primers and probe were designed according to Tan *et al.* [19]. The forward primer employed was 5′-GAGCTCTATAGGAGATAGTGTGAGTAGGG-3′, the reverse primer was 5′-ATGACTGCTCACCTGCGTGTT-3′ and the TaqMan probe used was 5′6-FAM-ACTTACCCA/ZEN/GGCCCTGCCAGCTCC-lowa Black FQ-3′. The viral RNA samples were extracted using QIAamp Viral RNA mini kit (QIAGEN, Hilden, Germany) according to the manufacturer’s instructions. The RT TaqMan real-time PCR assay was performed with the OneStep™Plus Real Time System (ABI, Carlsbad, USA) using TaqMan® Fast virus 1-step master mix (ABI, Carlsbad, USA) with cDNA synthesis by reverse transcription for 5 minutes at 50°C and subsequently amplified for 40 cycles at 95°C for 3 s, 60°C for 30 s.

### SDS-PAGE and Western Blot Analysis

RD cells were seeded at 7.5×10^5^ cells/well in a 6-well plate and followed by overnight incubation at 37°C in a CO_2_ incubator. Prior to infection, both RD cells and virus were pre-treated with various concentrations of the peptides for 1 hour. At 24-hour post-infection, the cells and viruses were harvested. The cells were lysed using 100 µl of ReadyPrep Sequential Extraction Kit Reagent 2 (Bio-Rad, USA). The protein concentration was determined using the MicroBCA protein assay (Pierce, Rockford, USA). An aliquot of 30 µg of each lysate was electrophoresed in a denaturing 12.5% polyacrylamide gel. The proteins were transferred onto a PVDF membrane (Millipore, Billerica, USA), and the membrane was subsequently blocked in 5% skimmed milk powder in phosphate buffered saline (PBS) with 0.05% Tween-20 for 1 hour in room temperature. The membrane was incubated with 1∶1000 diluted anti-EV-71 monoclonal antibody (Millipore, Billerica, USA) or 1∶1000 diluted β-actin antibody (Sigma, St. Louis, USA) for 1 hour at room temperature. After the membrane was washed, it was incubated with 1∶1000 diluted secondary antibody (HRP-conjugated rabbit anti-mouse antibody, Sigma, St. Louis, USA) for 1 hour at room temperature. The immunoblots were developed with the DAB substrate in stable peroxide substrate solution (Pierce, Rockford, USA).

### Mechanism of Action of SP40

RD cells were seeded at 1.5×10^4^ cells per well in a 96-well plate and incubated overnight at 37°C in a CO_2_ incubator before EV-71 infection with a MOI of 0.1 per well. Peptides were added at various concentrations under the following conditions: (i) Cell protection assay: RD cells were treated with various concentrations of peptide (50 µl/well). After 1 hour, the cells were washed twice with serum free medium and infected with 100 µl/well of EV-71 (1.5×10^4^ PFU/ml) for 1 hour. The medium containing virus was replaced with fresh maintenance medium and the viral titer was determined 24-hour post-infection using plaque assay. (ii) Post-infection assay: RD cells were first infected with EV-71 for 1 hour before addition of the peptide. The inocula were replaced with fresh maintenance medium containing various concentrations of peptide (50 µl/well) and the viral titer was determined 24 hours later. (iii) Virucidal assay: EV-71 (1×10^6^ PFU/ml) was treated with various concentrations of peptide in 100 µl of maintenance medium for 1 hour. The treated virus was then diluted 200-fold and used to infect cells for 1 hour. The diluted virus-peptide mixtures were replaced with fresh maintenance medium, and the plaque forming unit was determined 24 hours later. (iv) Viral attachment assay: RD cells in the chamber slide (Lab-tek, Rochester, USA) or a 96-well plate or a CellCarrier-96 optic black plate (Perkin-Elmer, Waltham, USA) were pre-treated with the peptide for 1 hour at 4°C and subsequently infected with EV-71 at the MOI of 100 for 1 hour at 4°C. The infected cells in the 96-well plate were washed twice to remove unbound viral particles, the attached EV-71 viral RNA were extracted and quantitated by the RT TaqMan Real-time PCR assay. The infected cells in the chamber slide and the CellCarrier-96 optic black plate were fixed with 4% paraformaldehyde and permeabilized with 0.25% Triton-X. The slide was first blocked with Image-iT™ FX Signal Enhancer (Invitrogen, San Diego, USA) for 1 hour. Subsequently, anti-Enterovirus 71 monoclonal antibody (Millipore, Billerica, USA) was added into the cell-coated well followed by 1∶200 diluted Alexa Fluor 488 anti-mouse IgG (Invitrogen, USA) as a secondary antibody. The nuclei of the RD cells were stained with 4,6-diamidino-2-phenylindole (DAPI). The slide was observed under Nikon Eclipse TE2000-E Fluorescence Microscope and the CellCarrier-96 optic black plate was analyzed by Cellomics High Content Screening ArrayScan VTI (Thermo Fisher Scientific, USA) using Spot Detector Bio-Application. The number of spots per field was determined.

### Cytotoxicity Assay

Peptide cytotoxicity was determined by a commercially available assay (Celltiter 96 AQueous One Solution Cell Proliferation Assay Reagent, Promega, Madison, WI) following the manufacturer’s instructions. Briefly, RD cells (1.5X10^4^ cells/well) were seeded in a 96-well plate, and the plate was incubated for 24 hours at 37°C. An aliquot of 20 µl of the medium containing the desired concentration of the SP40 peptide was added to the cells. The final concentration of DMSO for all peptide dilutions was adjusted to 0.6% to eliminate the effect of DMSO variation on cell cytotoxicity. After incubation of the cells in the presence of the SP40 peptide overnight at 37°C, 20 µl of the 96 AQ_queous_ One Solution cell proliferation assay reagent were added to each well. The plate was then incubated for 2 hours at 37°C and the absorbance at 490 nm was determined with a 96-well plate reader.

### Sequence Alignment and Structure Homology Prediction

The genome sequences of the EV-71 strain 41 (GenBank accession number: AAK13008) and the Mahoney poliovirus strain (GenBank accession number:1PO2_1) were searched using the NCBI Protein website (http://www.ncbi.nlm.nih.gov/protein/). The sequence of the EV-71, VP1 protein was aligned using Clustal W2 program (http://www.ebi.ac.uk/Tools/Clustalw2/index.html). The three-dimensional structure of the VP1-VP4 protein complex of Mahoney strain poliovirus (PDB ID: 1HXS) was selected to predict the possible position of SP40 by homology modeling.

## Results

### Screening of the VP1 Capsid Protein Peptide Library

**Figure 1:**
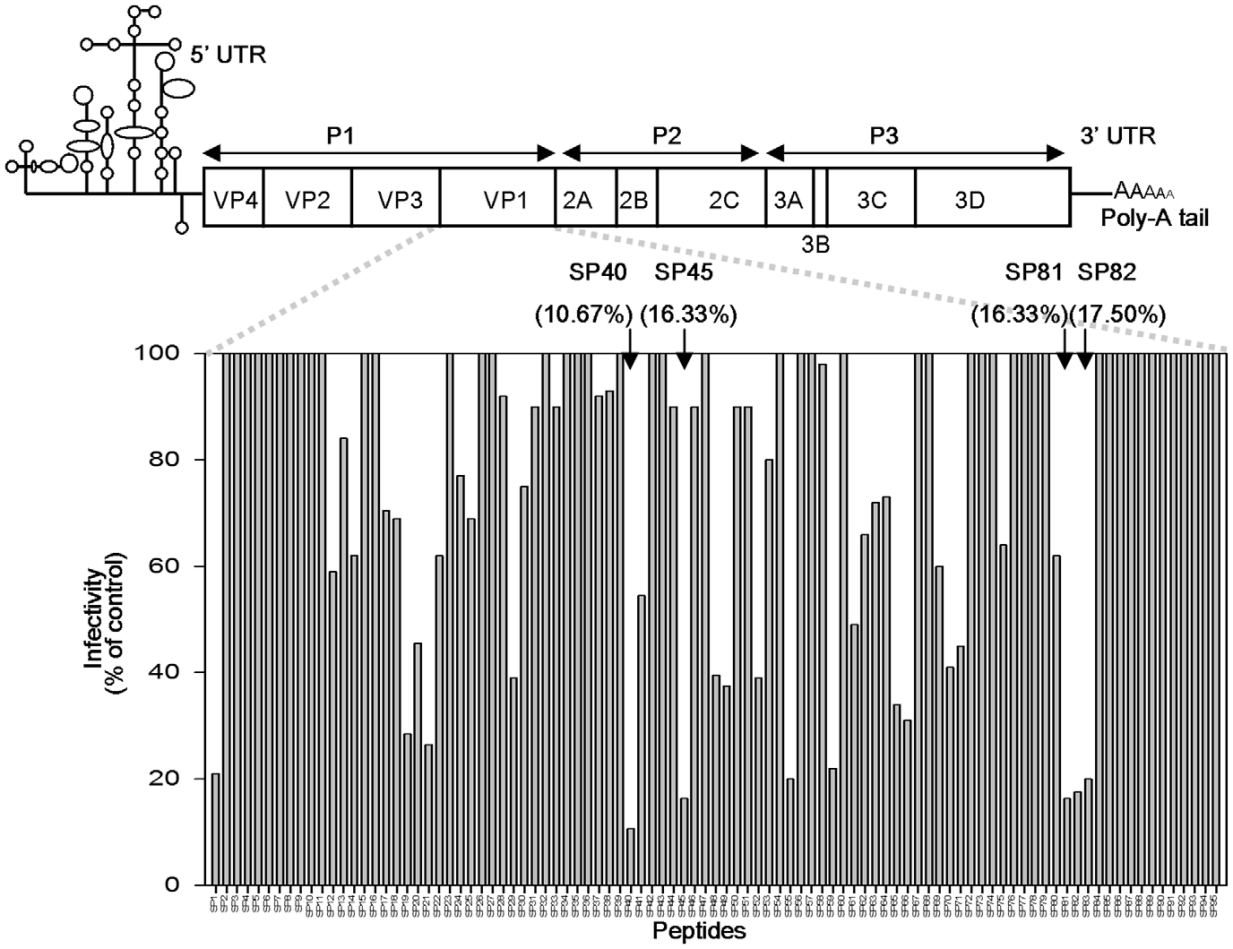
Identification of antiviral peptides. A library consisting of 95-overlapping peptides (15 mers) covering the entire EV-71 capsid protein, VP1 was synthesized. Each peptide was screened at a presumptive concentration of 100 µM for its ability to inhibit EV-71 infection in plaque reduction assay. The top line showed a schematic representation of the EV71 genome, and the activities of the peptides were shown at the bottom. Arrows denote the four peptides that were positive in the assay.

To test the potential of using peptides as an antiviral agent, a library of 15-mer peptides (purity at 60–65%) corresponding to residues 1 to 297 of the VP1 capsid protein and overlapping with each other by 12 residues (6 residues on the C-terminal and N-terminal, respectively) were synthesized. All the 95-overlapping peptides were evaluated for their ability to reduce cytopathic effects caused by EV-71 in RD cells, followed by plaque reduction assay as described in the materials and methods. The criterion for designating a peptide as antiviral is the inhibition of at least 80% of plaque formation at a concentration of 100 µM. Fig. 1 shows the locations of the peptides in the EV-71 genome and their antiviral activities in the initial screen. Of the 95-overlapping peptides in the library, there were four peptides, designated as SP40, SP45, SP81 and SP82 that were found to exhibit inhibitory effects against EV-71 plaque formation at 89.3%, 83.7%, 83.7%, and 82.5%, respectively (Fig. 1). The SP40 peptide was selected for further analysis as SP40 showed the highest inhibition of both cytopathic effect and plaque reduction. The amino acid sequence of the SP40 peptide was highly conserved across all genotypes of EV-71 (Table S1). SP40 is a 15-mer peptide (Ac-QMRRKVELFTYMRFD-NH_2_) spanning from position 118 to 132 in the VP1 capsid region. A scrambled-SP40 peptide, designated as SP40X (Ac-REFTMKRMVLFRQDY-NH_2_), was synthesized and used as a control throughout the experiments.

### Synergistic Antiviral Activities of the SP40 Peptide with SP81

To investigate whether the SP40 peptide exhibited synergistic antiviral activities with other peptides, the SP40 peptide was mixed with SP45, SP55 and SP81 (<70% purities) to a final concentration of 200 µM. Our results demonstrated that the SP40 peptide could best exhibit synergistic antiviral activities with the SP81 peptide to achieve a total viral RNA inhibition of 99.7% when compared to 92.0% being exhibited by 200 µM of the SP40 peptide alone. However, the SP40 peptide had less synergistic effect when combined with the SP45 peptide or the SP55 peptide, with viral RNA inhibitions of 94.3% and 96.0%, respectively. When all the 4 peptides were combined, viral RNA inhibition achieved was at 98.8%.

### Antiviral Properties of SP40

**Figure 2:**
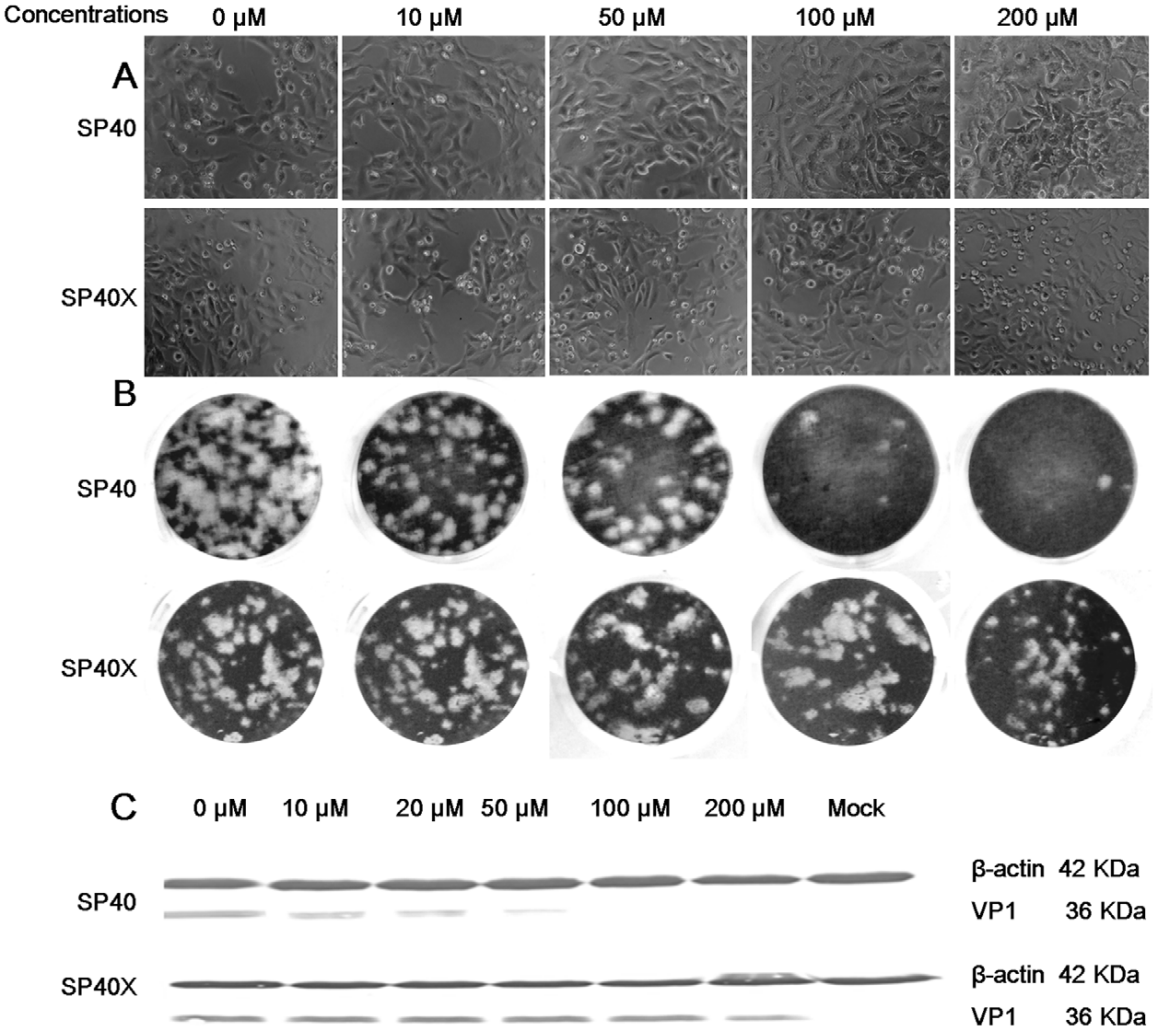
Inhibitory effects of SP40 and SP40X peptides in cytopathic effect, plaque formation and protein synthesis. (A) For cytopathic effect, EV-71 at a MOI of 0.1 was pre-incubated with peptides for 1 hour before infection of peptide-treated RD cells. The images were taken at 24-hour post-infection. (B) For plaque reduction assay, approximately 100 PFU of EV-71 were pre-incubated with peptides for 1 hour before infection of peptide-treated RD cells. The cells were fixed with 4% formaldehyde and stained with 0.5% crystal violet at 48-hour post-infection. (C) Western blot analysis of total protein isolated from virus-infected cells using the EV-71 monoclonal antibody (Millipore, Billerica, USA) and monoclonal anti β-actin antibody (Sigma, St. Louis, USA).

**Figure 3:**
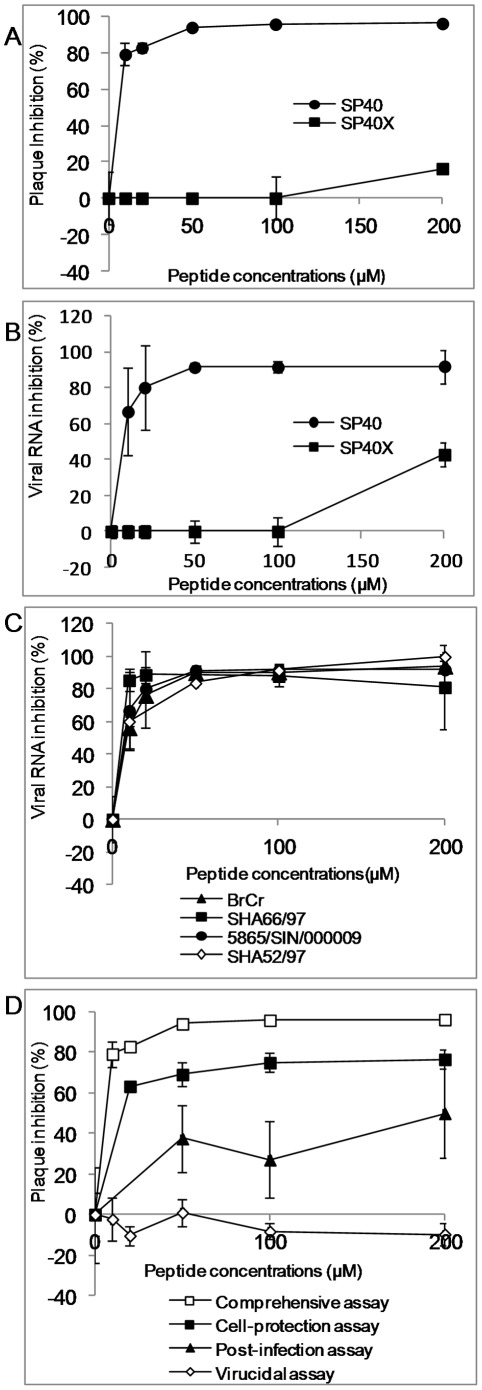
Antiviral activities of the SP40 and SP40X peptides. Both RD cells and EV-71 were separately pre-incubated with increasing concentrations of each peptide for 1 hour before viral inoculation. The inhibitory levels of the peptides were evaluated at 24-hour post-infection by (A) plaque assay and (B) RT TaqMan real-time PCR. (C) Antiviral properties of the SP40 peptide against different EV-71 strains in the comprehensive assay and (D) Mechanism of action studies: The SP40 peptide was added to EV-71 infection at different time points relative to viral inoculation as previously described in the Materials and Methods.

**Figure 4:**
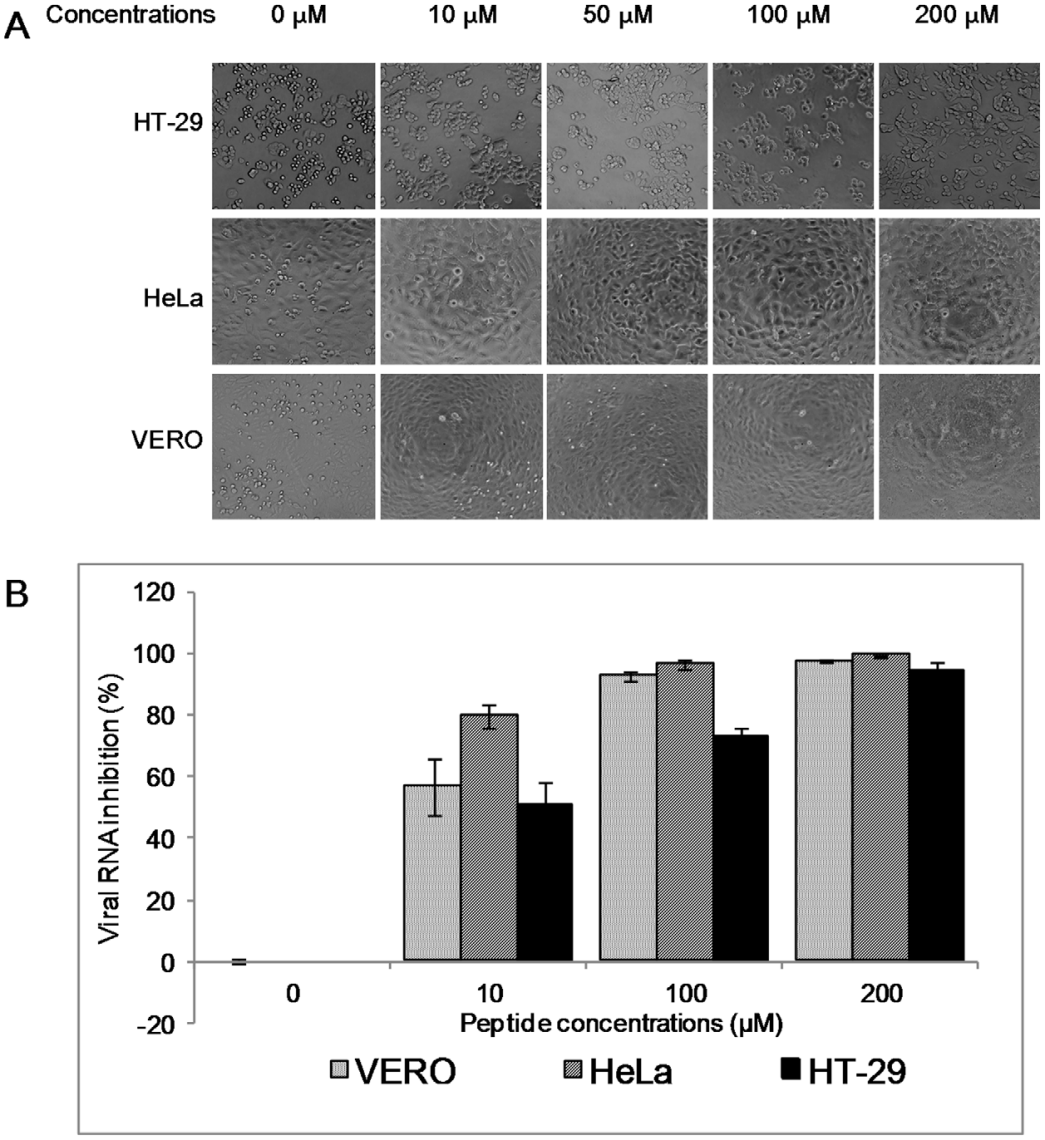
The antiviral activities of the SP40 peptide in various cell lines. Vero, HeLa and HT-29 cell lines were pre-treated with the SP40 peptide at various concentrations for 1 hour at room temperature before infection with EV-71 at a MOI of 0.1. (A) The viral induced cytopathic effects in various cell lines were observed 24-hour post-infection. (B) The viral RNA inhibition that were quantitated by RT TaqMan real-time PCR assay.

To further confirm that the SP40 peptide inhibited EV-71 infection in RD cells, the peptide was synthesized on a larger scale with >95% purity. The peptide was tested in the comprehensive assay against EV-71 at a MOI of 0.1. The results confirmed that SP40 inhibited viral plaque formation and RNA synthesis with inhibition levels achieved at 96.3%±0.8 and 92.0%±9.3, respectively when the peptide was applied at 200 µM (Fig. 2B, Fig.3A and 3B). The SP40 peptide also significantly reduced viral cytopathic effect and protein synthesis when tested in RD cells (Fig. 2A, Fig. 2C). To determine whether the amino acid sequence of the SP40 peptide is critical for the antiviral activities, the antiviral property of the scrambled SP40X peptide was evaluated. The data indicated that the SP40X peptide showed no significant inhibition of EV-71 infection at 200 µM. Two independent experiments confirmed that the SP40 peptide inhibited EV-71 strain 41 infection with an IC_50_ of 7.9 µM±3.5 (Table 1). Interestingly, the SP40 peptide also inhibited all three genotypes of EV-71 (genotypes A, B and C) with IC_50_ values ranging from 6–9.3 µM (Table 1, Fig. 3C). The SP40 peptide was found to inhibit EV-71 induced cytopathic effects and viral RNA synthesis in Vero, HeLa and HT-29 cell lines in a dose-dependent manner (Fig. 4A and 4B). The SP40 peptide also exhibited antiviral activities against CV-A16 and poliovirus type 1 with IC_50_ values of 6 µM±0.8 and 18.22 µM±10.4, respectively. However, the IC_50_ value against poliovirus type 1 was observed at a higher value (Table 1). Hence, we concluded that the SP40 peptide exhibited a broad-spectrum antiviral activity against all EV-71 genotypes as well as other enteroviruses in various cell lines.

**Table 1:** IC_50_ of the SP40 peptide against various enteroviruses.

EV-71 strains	Genotypes	Clinical manifestations	IC_50_ (µM)^b^
BrCr	A	Aseptic meningitis	9.3±2.5
SHA66/97	B3	HFMD^a^	6±0.7
41	B4	Fatal	7.9±3.5
SHA52	C2	HFMD	8.5±2.8
Coxsackievirus A16	–	HFMD	6±0.8
Poliovirus type 1	–	–	18.22±10.4

a HFMD denotes hand, foot and mouth disease.

b The IC_50_s are the mean ± standard deviations determined from at least two independent experiments.

### Mechanism of Action of SP40

We have found that the SP40 peptide exhibited the strongest inhibitory effect when tested in the comprehensive assay with an IC_50_ of 7.9 µM where both RD cells and EV-71 viral particles were first pre-treated with the SP40 peptide separately before infection (Fig. 3D). This suggested that the SP40 peptide could exert its inhibition at the viral binding step in EV-71 infection or it was virucidal to EV-71 viral particles.

To determine if the SP40 peptide could inactivate EV-71 viral particles, viral supernatant at a MOI of 10 was incubated with the SP40 peptide for 1 hour at room temperature, and subsequently diluted 200-folds and viable viral particles were quantitated by the plaque assay. The results demonstrated that the SP40 peptide could not inactivate EV-71 even when the peptide tested was present at a concentration as high as 200 µM. Hence, SP40 peptide was not virucidal to EV-71 viral particles (Fig. 3D).

**Figure 5:**
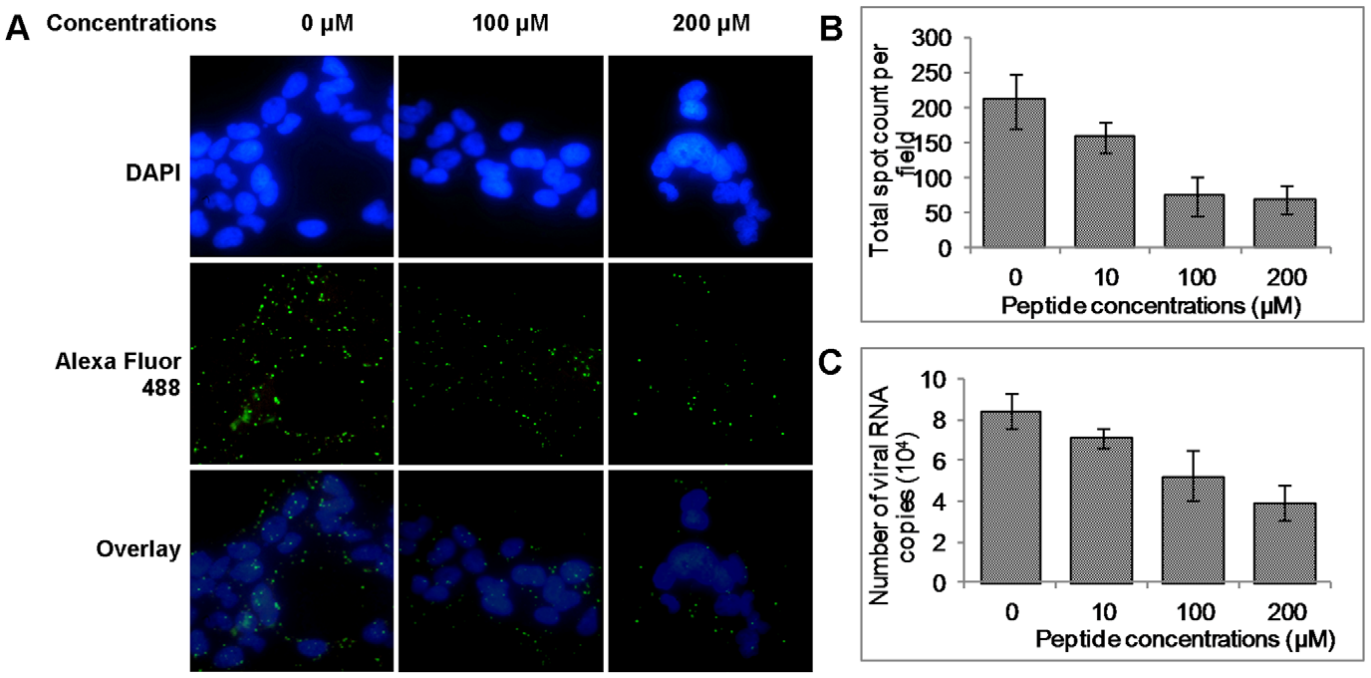
Viral attachment assay. (A) RD cells were grown in the chamber slides (Lab-tek, Rochester, USA) and incubated at room temperature for 1 hour with or without the SP40 peptide. This was followed by the incubation of the cells in the cold with EV-71 for 1 hour and washing off the unbound virions with PBS. RD cell monolayers were fixed with 4% paraformaldehyde and subsequently blocked with Image-iT™ FX Signal Enhancer (Invitrogen, San Diego, USA). The EV-71 particles were probed with anti-EV-71 monoclonal antibody (Millipore, Billerica, USA) and Alexa Fluor 488 anti-mouse IgG (Invitrogen, San Diego, USA). The nuclei were stained with DAPI for 7°minutes at room temperature. The images were obtained from the fluorescent microscopy. EV-71 viral particles and cell nuclei were shown in green and blue fluorescence, respectively. The number of virus particles that was attached to the cell surface were quantitated by (B) Cellomics HCS ArrayScan Spot Detector Bio-Application and (C) RT TaqMan real-time PCR assay

To elucidate whether the SP40 peptide could inhibit the viral binding step in EV-71 infection, RD cells were pre-incubated with the SP40 peptide for 1 hour at room temperature before EV-71 infection. The IC_50_ value observed was 15 µM (Fig. 3D). The data indicated that the SP40 peptide could disrupt or interfere the binding of the EV-71 viral particles to cells or it could interfere with post-binding steps. To address this, RD cells were pre-treated with various concentrations of the SP40 peptide at 4°C for 1 hour, followed by EV-71 inoculation at a MOI of 100 at 4°C. The EV-71 viral particles that were attached to the RD cell surface were determined by immunofluorescence assay as described in the Materials and Methods. As shown in Fig. 5A, the number of EV-71 viral particles (green fluorescence) attached to the RD cell surface was observed to be reduced when tested in the presence of the SP40 peptide. The results from the Cellomics HCS ArrayScan Spot Detector BioApplication assay showed that the number of viral particles that were attached to the cell surface were reduced from 210±39 viral spots per field to 68±20 viral spots per field in a dose dependent manner (Fig. 5B). Total viral RNA determined by real-time RT-PCR assay confirmed significant reduction of viral RNA following treatment with the SP40 peptide before infection with EV-71 at 4°C (Fig. 5C). To investigate if SP40 inhibited a post-binding step in EV-71 entry to the cells, RD cells were incubated with EV-71 for 1 hour at 4°C, followed by the addition of SP40. The cells were washed and immediately shifted to 37°C for 1 hour to allow post-binding event. No inhibition of plaque formation was observed (data not shown). The results demonstrated that the SP40 peptide disrupted the binding of the EV-71 viral particles to RD cells rather than at the EV-71 during post-binding stage.

The SP40 peptide was found to be non-inhibitory when added one hour after RD cells were infected with EV-71. The IC_50_ of SP40 peptide after 1 hour post-infection was established to be 200 µM (Fig. 3D).

### Residues Critical for Antiviral Activity of SP40

**Figure 6:**
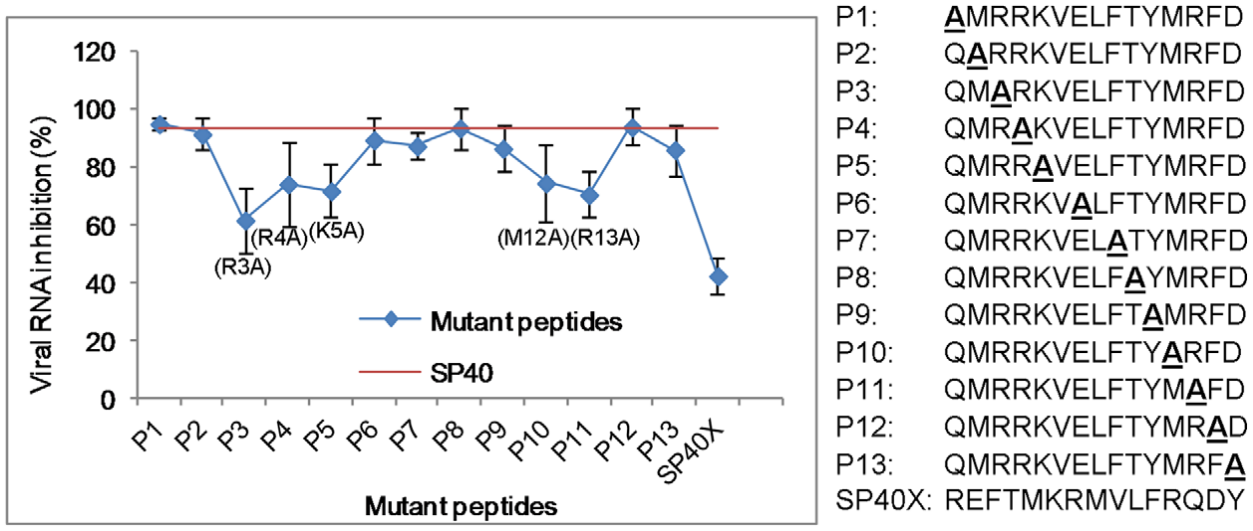
Alanine scanning analysis. Alanine scanning was performed on the SP40 peptide. Thirteen different peptides were synthesized by replacing one residue at a time with an A and their inhibitory effect was determined as described above. Activity of each peptide was compared with the wild-type SP40, which was represented by the red line. Numbers higher than the red line showed a gain of activity whereas a lower number represented a loss of activity.

To identify the residues in the SP40 peptide that are critical for antiviral activity, 13 peptides with alanine substitution in each of the amino acid position in the 15-mer SP40 peptide were synthesized. The inhibitory effects in RD cells at 200 µM of all peptides were evaluated. Reduction of viral RNA levels by each of the peptides being evaluated was summarized in Fig. 6. Substitution of the arginine residue at position 3 (P3) with alanine in the SP40 peptide was found to significantly decrease the inhibitory activity (from 95.9% to 61.8%) when compared to substitution at other positions. When positively charged arginine or lysine residues at positions 4, 5 and 13 (P4, P5 and P11) of the SP40 peptide were substituted with alanine, there were moderate losses of activities (from 95.9% reduced to 74.3%, 70.9% and 70.6%, respectively). With only one exception, substitution of the polar methionine residue at position 12 with alanine in the SP40 peptide also reduced the antiviral activity moderately to 74.7%. Alanine substitutions of amino acids at other positions of the SP40 peptide did not alter the antiviral activities when compared to the SP40 peptide. Our data indicated that the positively charged amino acids were critical for the antiviral activities of the SP40 peptide.

### Cytotoxicity Assay

To evaluate whether the SP40 peptide was cytotoxic to cells, RD cells were treated with increasing concentrations of the SP40 peptide from 0 µM to 280 µM, and cell viability was determined using CellTiter 96 AQueous One Solution Cell Proliferation Assay Reagent (Promega, Madison, WI). We found that the SP40 peptide was non-cytotoxic to RD cells when tested at the concentration of up to 280 µM. The SP40 peptide was also non-cytotoxic to HeLa, Vero, HT-29 cell lines (data not shown).

### Homology Modeling of EV-71

**Figure 7:**
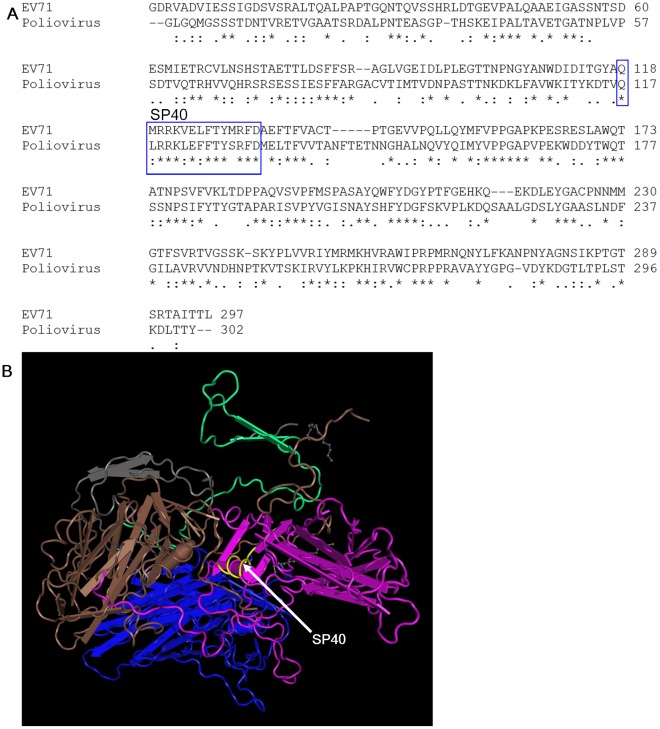
Proposed locations of the SP40 peptide based on sequence alignment and molecular modeling of poliovirus structure. (A) The EV-71 strain 41 was aligned with Mahoney poliovirus strain using Clustal W2 program and (B) The molecular structure of poliovirus VP1, VP2, VP3, and VP4 is represented by purple, blue, brown and green, respectively. The SP40 sequence is indicated in yellow.

The EV-71 VP1 amino acid sequence was aligned with Mahoney poliovirus using Cluster W2 program and the three dimensional structure of the EV-71 capsid protein based on the poliovirus model was analyzed by the NCBI Cn3D 4.3 software (Fig. 7). The amino acid sequence of the SP40 in the 3D structure is indicated in yellow. The 3D-homology structure of EV-71 indicated that part of the SP40 peptide was exposed on the surface.

## Discussion

A Pepscan strategy was employed to screen 95-overlapping synthetic peptides corresponding to the VP1 capsid protein for antiviral activity against EV-71. Four peptides SP40, SP45, SP81 and SP82 were found to exhibit significant antiviral activities. The SP40 peptide was selected for further investigation as the amino acid sequence of SP40 is highly conserved across all EV-71 genotypes and sub-genotypes. Our results demonstrated that the SP40 peptide inhibited EV-71 infection in a dose-dependent manner corresponding to the reduction of viral RNA, VP1 protein and plaque formation. The IC_50_ values reported in our studies ranged from 6–9.3 µM against all representative strains of EV-71 genotypes A, B and C. Interestingly, the SP40 peptide also inhibited CV-A16 and poliovirus type 1 infection *in vitro*, implying that the SP40 peptide could function as a broad-spectrum antiviral agent. However, a higher concentration of SP40 peptide was required to block poliovirus type 1 infection. This could be due to the high degree of dissimilarity of the amino acid sequence present in EV-71 and poliovirus.

The possible mechanism of action of the SP40 peptide could be either through direct viral inactivation or it could block viral attachment and entry. Our data confirmed that the SP40 peptide was not virucidal, but it blocked viral attachment to the cell-surface and hence prevented EV-71 infection. Our immunofluorescence assay and Cellomics HCS ArrayScan showed the number of viral particles attached to the cell surface was reduced significantly when the RD cells were pre-treated with the SP40 peptide before addition of virus at 4°C. The results indicated that the SP40 peptide probably first interacted with a cell-surface receptor and subsequently prevented EV-71-cellular receptor interactions. However, the SP40 peptide lost its antiviral activity when the peptide was added 1 hour after EV-71 infection.

Previous studies have shown that peptides could play a significant role in surface protein-protein interactions and could exert inhibitory activities against viruses like influenza virus [20], Herpes Simplex virus-1 [21], [22], Hepatitis B virus [15], Hepatitis C virus [14], [23], HIV-1 [12], Dengue virus and West Nile virus [24]. Although the EV-71 capsid protein VP1 has been reported to be responsible in mediating viral adsorption and uncoating process, little information is available about the molecular interactions of EV-71 and cell receptors [25]. Recently, Chen *et al.* [26] had identified several amino acid residues present in the EV-71 capsid protein VP1 that were critical for the molecular interaction between EV-71 and the SCARB2 receptor. These amino acid residues were found within the residues 152–236 of the VP1 protein. None of the amino acids identified was mapped within the SP40 peptide amino acid sequence. This finding suggested that the SP40 peptide probably did not interact with the SCARB2 receptor.

We have demonstrated the importance of the SP40 amino acid sequence for antiviral activities by comparison with a peptide carrying scrambled sequence. The inhibitory effect of the scrambled peptide, SP40X, was significantly lower (at 42.5%) than the effect observed with the SP40 peptide. Since the amino acid sequence of the SP40 peptide was highly conserved across all EV-71 genotypes and was exposed on the surface, this sequence might carry important motif/domain that interacted with an unidentified cell-surface receptor. The SP40 peptide could prevent viral attachment by interacting with cell receptors present on the surfaces of the RD cells, thereby blocking the availability of the receptor for attachment of the EV-71 viral particles. The observation of a significantly reduced IC_50_ value when RD cells were pre-treated with SP40 before EV-71 infection strongly supports this view. The cellular receptor that the SP40 peptide interacted with remained unknown. However, the SP40 peptide could also inhibit CV-A16 and poliovirus type 1 infections *in vitro*, indicating that the SP40 peptide could interact with a common receptor that was probably shared by these viruses.

Since the positively charged amino acids were critical for antiviral activities, the SP40 peptide could interact with cell surface receptors through electrostatic charge interactions. The antiviral activity of the SP40 peptide was not restricted to a specific cell type, but it could block EV-71 infection in different cell lines. This indicated that the SP40 peptide was probably interacting with the receptor that was commonly expressed in most cell types. Interestingly, cell surface glycosaminoglycans are present ubiquitously on the surface of most animal cells and in the extracellular matrix [27]. The presence of several arginine residues in SP40 draws similarity to the antiviral peptide displaying positively charged poly-arginine residues against herpes simplex virus-I (HSV-I) [22]. The antiviral property of the poly-arginine peptide against HSV-I infection in mice was due to an interaction with heparan sulfate. Sequence analysis of the SP40 peptide revealed that it consisted of heparan sulfate glycosaminoglycan specific binding domains (G_1_RRRRS_6_ and R_28_KVR_31_) present in bovine and human lactoferrins [28]–[30]. Several studies have reported that lactoferrin was able to bind to ligands such as heparan sulfate and chondroitin sulfate [28], [31], [32]. It is possible that through this interaction lactoferrin was able to inhibit EV-71 infection [33]. These findings suggested that the SP40 peptide could have interacted with cell surface glycosaminoglycans and prevented EV-71 attachment.

This is the first time that a small novel viral-based peptide (15-mer) derived from VP1 is reported to exhibit antiviral activities against all genotypes and sub-genotypes of EV-71 infection *in vitro*. The development and use of antivirals like enviroxime [34], pleconaril [35], nucleoside analog ribavirin [36] and 3C protease inhibitors [37] for treating enteroviral infection showed variable efficacies against the neurotrophic EV-71 virus [38]. Our results showed that some EV-71 strains were even resistant to ribavirin at 800 µM (unpublished data), this contradicts with the finding of an IC_50_ of 266 µM reported by Li *et al.* [36]. This indicated that ribavirin might not serve as an effective antiviral agent against all EV-71 strains. Other antiviral agents like the viral capsid-binding pyridyl imidazolidinones were found to be ineffective when a single amino acid mutation occurred at position 192 of the hydrophobic pocket of the VP1 capsid protein [16]. The SP40 peptide was found to exhibit very similar antiviral properties with bovine and human lactoferrins which were predicted to prevent viral attachment, possibly by blocking an unknown cellular receptor [33], [39]. However, the exact antiviral mechanism of lactoferrin remains to be determined and the SP40 peptide reported in our study has an even lower IC_50_ value at 15 µg/ml when compared to the IC_50_ value of bovine lactoferrin at 34.5 µg/ml [33] or human lactoferrin at 103.3–185.0 µg/ml [39], [40]. Thus, SP40 is a good antiviral candidate.

The peptides that blocked the SCARB2 receptor could also be developed as antiviral agents. Chen *et al.* [26] have discovered the amino acid residues in the VP1 capsid protein that are critical for SCARB2 receptor binding. Interestingly, the amino acids in VP1 that are critical for SCARB2 binding were found in the SP45, SP55, and SP81 peptides. The amino acid residues that are important for SCARB2 binding in the SP45, SP55 and SP81 peptides were illustrated in Fig. S1. These peptides were able to inhibit EV-71 infections in a dose-dependent manner with no cytotoxicity to the RD cells (unpublished data). Strong synergistic antiviral activities were observed between the SP40 and the SP81 peptides in RD cells. Since the amino acids critical for binding to SCARB2 were not present in the SP40 peptide, the data suggested that the SP40 peptide could have interacted with a different receptor compared with the SP81 peptide. The additive effects of these two peptides could have significantly reduced the availability of receptors for viral attachment.

Inhibition of viruses at the stage of viral attachment provides a target for therapeutic intervention. Therapeutic peptides have become an attractive tool in drug discovery due to their active regulatory role in the biological system and their extreme high specificity of recognition. The best characterized therapeutic peptide inhibitor is Enfuvirtide (fusion inhibitor) which mimics the N terminal sequence in HIV fusion protein, gp41 [12]. Using peptides as therapeutic agents offer some significant advantages over small chemical molecules or large therapeutic antibodies. A major advantage of peptides is their small size and their high activity and specificity when compared to the antibodies. Peptides are better candidates to inhibit protein-protein interactions that comprise a surface area often too large to be inhibited by small chemical molecules. Peptides accumulate in lesser quantity in tissues, and have very low cell toxicity when compared to small synthetic molecules [41]. Antimicrobial peptides such as lactoferrin, human β-defensin-2 and dermaseptins have been reported to exhibit antiviral properties against various viruses [28], [42]–[46]. Therefore, therapeutic peptides have some advantages over the smaller chemical compounds as antiviral agents.

Since the SP40 peptide works at a very low micromolar concentration and is non-cytotoxic to RD cells, it is potentially an excellent candidate for further development as an antiviral agent. The SP40 peptide was effective when administered before EV-71 infection and could be considered as an excellent candidate for prophylactic intervention. The exact cellular receptor(s) that the SP40 peptide interacted with still remained unknown. Further *in vivo* studies are needed for development of the SP40 as an antiviral agent. Although a major disadvantage of peptides is their low bioavailability due to their rapid degradation in the gastrointestinal system, new formulations, such as the D-isomer peptide and other delivery options are being developed to circumvent these disadvantages [41].

## Supporting Information

Figure S1 **Diagrammatic illustration of EV-71 VP1 secondary structure.** Cylinder and arrow represent α-helix structure and β-sheet, respectively. The effects of mutations examined as to which amino acids were interacting with the SCARB2 receptor was marked as follows: Filled circle indicates those most effective residues for viral binding and infection; open circle indicates partial effective residues [26]. The amino acid sequences of SP40, SP45, SP55 and SP81-83 were shown to correspond to their secondary structure and location within the VP1 capsid protein.
(TIF)

Table S1 **VP1 protein sequences among enteroviruses.**
(DOC)
