# Supplementary figures and images for: Inhibition of Enterovirus 71 (EV-71) Infections by a Novel Antiviral Peptide Derived from EV-71 Capsid Protein VP1
Source: PLoS One. 2012 May 1;7(5):e34589. doi: 10.1371/journal.pone.0034589 (PMC3341398; doi:10.1371/journal.pone.0034589)

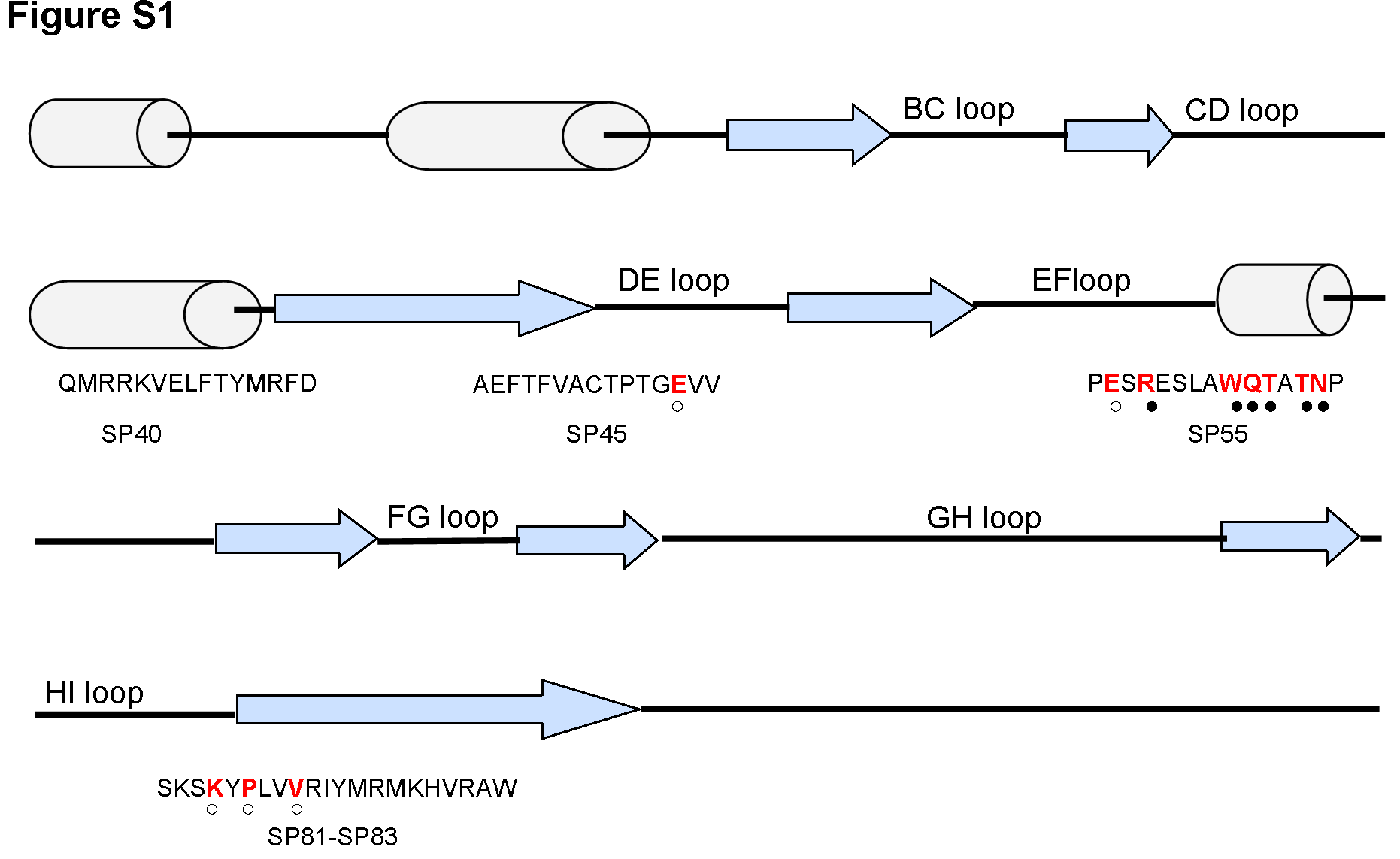

Supplement: Figure S1 — Diagrammatic illustration of EV-71 VP1 secondary structure. Cylinder and arrow represent α-helix structure and β-sheet, respectively. The effects of mutations examined as to which amino acids were interacting with the SCARB2 receptor was marked as follows: Filled circle indicates those most effective residues for viral binding and infection; open circle indicates partial effective residues [26]. The amino acid sequences of SP40, SP45, SP55 and SP81-83 were shown to correspond to their secondary structure and location within the VP1 capsid protein. (TIF) [file pone.0034589.s001.tif]
