# Supplementary material for: Inhibition of Enterovirus 71 (EV-71) Infections by a Novel Antiviral Peptide Derived from EV-71 Capsid Protein VP1
Source: PLoS One. 2012 May 1;7(5):e34589. doi: 10.1371/journal.pone.0034589 (PMC3341398; doi:10.1371/journal.pone.0034589)
Supplement: Table S1 — VP1 protein sequences among enteroviruses. (DOC) [file pone.0034589.s002.doc]

**Table S1. VP1 protein sequences among enteroviruses**

| Enteroviruses | Genogroups | SP40 Peptide sequence^a^ | Accession Number |
| --- | --- | --- | --- |
| BrCr | A | QMRRKVELFTYMRFD | AB204852 |
| 4F-AUS-99 | B1 | QMRRKVELFTYMRFD | AF376105 |
| 7423/MS*/*87 | B2 | QMRRKVELFTYMRFD | U22522 |
| SHA66/97 | B3 | QMRRKVELFTYMRFD | AM396586 |
| SHA63/97 | B3 | QMRRKVELFTYMRFD | AM396588 |
| 26/M/AUS/4/99 | B3 | QMRRKVELFTYMRFD | EU364841 |
| UH1/97 | B4 | QMRRKVELFTYMRFD | AM396587 |
| 5865/SIN/000009 | B4 | QMRRKVELFTYMRFD | AF316321 |
| SHA89/97 | B4 | QMRRKVELFTYMRFD | AJ586873 |
| 5511-SIN-00 | B5 | QMRRKVELFTYMRFD | DQ341364 |
| NUH0083/SIN/08 | B5 | QMRRKVELFTYMRFD | FJ461781 |
| 1M/AUS/12/00 | C1 | QMRRKVELFTYMRFD | AF376098 |
| 804-NO-03 | C1 | QMRRKVELFTYMRFD | DQ452074 |
| J115-MAL-01 | C1 | QMRRKVELFTYMRFD | DQ341360 |
| 5M/AUS/5/99 | C2 | QMRRKVELFTYMRFD | AF376106 |
| Tainan4643/98 | C2 | QMRRKVELFTYMRFD | AF304458 |
| NCKU9822/98 | C2 | QMRRKVELFTYMRFD | AF136379 |
| 06-KOR-00 | C3 | QMRRKVELFTYMRFD | DQ341355 |
| 03-KOR-00 | C3 | QMRRKVELFTYMRFD | DQ341356 |
| Fuyang-0805 | C4 | QMRRKVELFTYMRFD | FJ439769 |
| SHZH03 | C4 | QMRRKVELFTYMRFD | AY465356 |
| N3340-TW-02 | C4 | QMRRKVELFTYMRFD | EU131776 |
| E2005125-TW | C5 | QMRRKVELFTYMRFD | EF063152 |
| 2007-07364 | C5 | QMRRKVELFTYMRFD | EU527983 |
| CV-A16 | - | Q **L**RRK**C**ELFTYMRFD | AJ238454 |
| CV-A12/Texas | - | Q **L**RRK**L**E **I** FTYMRFD | AY421768 |
| CV-B3 | - | Q **L**RRK**L**E **F**FTY**V**RFD | M16572 |
| CV-B4 strain E4 | - | Q**L**RRK**M**E**M**FTY**I** R**C**D | AF311939 |
| Poliovirus (Mahoney) | - | Q **L**RRK**L**E **F**FTY**S** RFD | V01148 |

^a^ Bold-Underlined amino acids represent changes from the original SP40 peptide sequence.
